# Aktuelle dermatologische Leitlinien in Deutschland: eine Auswahl klinisch relevanter Empfehlungen

**DOI:** 10.1007/s00105-025-05601-1

**Published:** 2025-10-20

**Authors:** Marie Anne Pradeau, Maria Kinberger, Christos C. Zouboulis, David Chromy, Joachim Jackowski, Knut Brockow, Franziska Ruëff, Alexander Nast

**Affiliations:** 1 Klinik für Dermatologie, Venerologie und Allergologie, Division of Evidence-Based Medicine (dEBM), Charité – Universitätsmedizin Berlin, corporate member of Freie Universität Berlin and Humboldt-Universität zu Berlin, Charitéplatz 1, 10117 Berlin, Deutschland; 2 Hochschulklinik für Dermatologie, Venerologie und Allergologie, Immunologisches Zentrum, Städtisches Klinikum Dessau, Medizinische Hochschule Brandenburg Theodor Fontane und Fakultät für Gesundheitswissenschaften Brandenburg, Dessau, Deutschland; 3 Hidradenitis Suppurativa Foundation e. V., Dessau, Deutschland; 4 Universitätsklinik für Dermatologie, Medizinische Universität Wien, Wien, Österreich; 5 Abteilung für Zahnärztliche Chirurgie und Poliklinische Ambulanz, Department für ZMK-Heilkunde, Fakultät für Gesundheit, Universität Witten/Herdecke, Alfred-Herrhausen-Str. 45, 58455 Witten, Deutschland; 6 Klinik und Poliklinik für Dermatologie und Allergologie am Biederstein, Technische Universität München, München, Deutschland; 7 Klinik und Poliklinik für Dermatologie und Allergologie, Klinikum der Universität München, München, Deutschland

**Keywords:** Entscheidungshilfen, Dermatologie, Diagnostik, Haut, Therapie, Decision aids, Dermatology, Diagnosis, Skin, Therapy

## Abstract

Medizinische Leitlinien dienen als systematische Entscheidungshilfen, um eine evidenzbasierte und angemessene ärztliche Vorgehensweise bei bestimmten Krankheitsbildern zu gewährleisten. In Deutschland werden dermatologische Leitlinien unter der Federführung der Deutschen Dermatologischen Gesellschaft (DDG) und des Berufsverbandes der Deutschen Dermatologen (BVDD) entsprechend den methodischen Vorgaben der Arbeitsgemeinschaft der Wissenschaftlichen Medizinischen Fachgesellschaften (AWMF) entwickelt. Im Zeitraum 2023 bis 2024 wurden verschiedene Leitlinien mit Bezug zur Dermatologie überarbeitet oder neu herausgegeben. Dazu gehören unter anderem die Leitlinie zur Diagnostik und Therapie der epidermalen Nekrolyse (Stevens-Johnson-Syndrom und toxische epidermale Nekrolyse), der atopischen Dermatitis, der Hidradenitis suppurativa/Acne inversa, der kutanen Lyme-Borreliose, der primären Hyperhidrose, der zirkumskripten Sklerodermie, zum Screening auf anale Dysplasien und Analkarzinome bei HIV(humanes Immundefizienzvirus)-Infizierten, zu Aphthen und aphthösen Läsionen der Mund- und Rachenschleimhaut, zur allergologischen Diagnostik von Überempfindlichkeitsreaktionen auf Arzneimittel. Darüber hinaus wurden Aktualisierungen zu den Themen Basalzellkarzinom der Haut, analer Pruritus, Analekzem, aktinische Keratose und Plattenepithelkarzinom der Haut, Bienen- und Wespengiftallergie sowie Handekzem veröffentlicht. Diese Übersicht fasst die wichtigsten Empfehlungen und Neuerungen der aktuellen dermatologischen Leitlinien zusammen.

## Abstract (en)

Medical guidelines serve as systematic decision-making aids to ensure evidence-based and appropriate medical management of specific diseases. In Germany, dermatological guidelines are developed under the leadership of the German Dermatological Society (DDG) and the Professional Association of German Dermatologists (BVDD) following the methodology of the Working Group of Scientific Medical Societies (AWMF). In the period 2023–2024, various guidelines in the field of dermatology were revised or newly issued. These include guidelines on the diagnosis and treatment of epidermal necrolysis (Stevens–Johnson syndrome and toxic epidermal necrolysis), atopic dermatitis, hidradenitis suppurativa/acne inversa, cutaneous Lyme borreliosis, primary hyperhidrosis, circumscribed scleroderma, screening for anal dysplasia and anal carcinoma in HIV-infected individuals, aphthae and aphthous lesions of the oral and pharyngeal mucosa, and allergological diagnostics of hypersensitivity reactions to drugs. Additionally, updates were published on basal cell carcinoma of the skin, anal pruritus, anal eczema, actinic keratosis and squamous cell carcinoma of the skin, bee and wasp venom allergy, and hand eczema. This review summarizes the key recommendations and recent updates in dermatological guidelines.

Medizinische Leitlinien sind systematisch entwickelte Empfehlungen, die Ärztinnen und Ärzte bei der Entscheidungsfindung zu spezifischen gesundheitlichen Fragestellungen unterstützen. Laut der Bundesärztekammer dienen sie der standardisierten Patientenversorgung, unterscheiden sich jedoch von Richtlinien, die durch rechtlich legitimierte Institutionen festgelegt und verbindlich sind. Verstöße gegen Richtlinien können zu Sanktionen führen.

Die Deutsche Dermatologische Gesellschaft (DDG) erarbeitet in Zusammenarbeit mit dem Berufsverband der Deutschen Dermatologen (BVDD) kontinuierlich neue dermatologische Leitlinien und aktualisiert bestehende Empfehlungen. Zudem beteiligt sich die DDG an interdisziplinären Leitlinien anderer Fachgesellschaften.

Die Methodik der Leitlinienentwicklung folgt den Vorgaben der Arbeitsgemeinschaft der Wissenschaftlichen Medizinischen Fachgesellschaften (AWMF), wobei die Leitlinien je nach methodischer Stringenz in die Stufen S1 bis S3 eingeteilt werden (Tab. 1; [9]).

**Tab. 1:** Übersicht der Leitlinienentwicklungsstufen S1 bis S3. (Nach [9])

*S3*	Konsens- und evidenzbasierte Leitlinie	Repräsentatives Gremium; systematische Recherche, Auswahl und Bewertung der Literatur; strukturierte Konsensfindung
*S2e*	Evidenzbasierte Leitlinie	Systematische Recherche, Auswahl, Bewertung der Literatur
*S2k*	Konsensbasierte Leitlinie	Repräsentatives Gremium, strukturierte Konsensfindung
*S1*	Handlungsempfehlungen von Expertengruppen	Konsensfindung in einem informellen Verfahren

Diese Übersicht stellt eine Auswahl der in den Jahren 2023 und 2024 veröffentlichten oder aktualisierten deutschen dermatologischen Leitlinien sowie interdisziplinäre Leitlinien mit dermatologischer Relevanz vor.

## Leitlinien unter Federführung der Deutschen Dermatologischen Gesellschaft

### S3-Leitlinie „Diagnostik und Therapie der epidermalen Nekrolyse (Stevens-Johnson-Syndrom und toxisch epidermale Nekrolyse) SJS-TEN“ [11, 13]

Die S3-Leitlinie zur Diagnostik und Therapie der epidermalen Nekrolyse (EN) bietet umfassende Empfehlungen für das Management dieser schweren Hauterkrankung. EN ist häufig durch Medikamente (65–85 % aller Fälle bei Erwachsenen) oder Infektionen ausgelöst.

Typische Befunde sind erythematöse, teils kokardenartige Makulae und Epidermolysen. Das Nikolski-Zeichen, bei dem sich die Haut bei leichtem Druck ablöst, ist ein wichtiges klinisches Zeichen. Zudem sind schmerzhafte Erosionen der Mundschleimhaut, Lippen, oberen Atemwege, Konjunktiven und Genitalschleimhäute typisch. Bei Erstdiagnose einer EN soll innerhalb von 24 h eine ophthalmologische und dermatologische Untersuchung erfolgen.

Zur Diagnosesicherung dient eine Hautbiopsie. Diese sollte aus dem Übergangsbereich zwischen betroffener und gesunder Haut entnommen werden. Zudem ist eine SCORTEN(Score for Toxic Epidermal Necrolysis)-Erhebung an den Tagen 1, 3 und ggf. Tag 5 zur Prognoseeinschätzung wichtig.

Die Leitlinie gibt Hilfestellungen zur Identifikation eines möglichen medikamentösen Auslösers. Mögliche Auslöser sollen abgesetzt werden. Arzneimittel, die kürzer als 4 oder länger als 28 Tage eingenommen wurden, sind als Auslöser wenig wahrscheinlich und können in der Regel weiter verabreicht werden.

Die vorhandene Evidenz zur medikamentösen Therapie ist weiterhin sehr begrenzt, und die Empfehlungen zur immunmodulierenden Therapie wurden sämtliche als offene „Kann“-Empfehlung formuliert. Es kann die systemische Gabe von Glukokortikosteroiden, Ciclosporin A („off-label“) oder Etanercept („off-label“) erwogen werden. Die alleinige Behandlung mit intravenösen Immunglobulinen („off-label“), auch in Kombination mit systemischen Glukokortikosteroiden, kann basierend auf der eingeschlossenen Evidenz noch nicht abschließend beurteilt werden. Die supportive Therapie umfasst Flüssigkeits- und Elektrolytsubstitution, Schmerzmanagement und Infektionsprävention. Eine sorgfältige Wundversorgung ist essenziell, um die Heilung zu fördern und Komplikationen zu vermeiden.

Die interdisziplinäre Zusammenarbeit verschiedener Fachrichtungen – einschließlich Dermatologie, Intensivmedizin und Ophthalmologie – ist für eine optimale Patientenversorgung unerlässlich.

### S3-Leitlinie „Atopische Dermatitis“ [15, 16]

Aufbauend auf der europäischen EuroGuiDerm-Leitlinie wurde die deutschsprachige Leitlinie zur atopischen Dermatitis (AD) umfassend aktualisiert und auf S3-Niveau angehoben.

Das interdisziplinäre Leitliniengremium empfiehlt als Basistherapie neben der Meidung individueller Triggerfaktoren die regelmäßige Anwendung von Emollienzien.

Bei leichten bis moderaten Ekzemen soll zudem eine antiinflammatorische Therapie mit topischen Glukokortikoiden eingesetzt werden, die nach Abheilung der Läsionen als proaktive Therapie fortgeführt werden kann. In sensiblen Arealen wie dem Gesicht, den intertriginösen Bereichen und im Anogenitalbereich sollen antiinflammatorisch bevorzugt topische Calcineurininhibitoren eingesetzt werden, da diese keine Hautatrophie induzieren.

Bei moderaten bis schweren Verläufen kann eine Phototherapie (Schmalspektrum-UVB, UVA1) eingesetzt werden, längere oder repetitive Zyklen sind jedoch wegen des Hautkrebsrisikos zu vermeiden.

Für Patient*innen, die eine Systemtherapie benötigen, empfiehlt die Leitlinie die Biologika Dupilumab und Tralokinumab sowie die JAK(Januskinase)-Inhibitoren Upadacitinib, Abrocitinib und Baricitinib mit der höchsten Empfehlungsstärke. Da Lebrikizumab und Nemolizumab zum Zeitpunkt der Veröffentlichung der Leitlinie noch nicht zugelassen waren, gibt es für diese 2 Wirkstoffe bislang noch keine Empfehlung in der Leitlinie. Eine Off-label-Behandlung mit Methotrexat sollte nur eingesetzt werden, wenn die zuvor genannten Substanzen nicht wirksam oder kontraindiziert sind.

Ciclosporin A erhielt aufgrund des ungünstigeren Sicherheitsprofils lediglich eine offene „Kann erwogen werden“-Empfehlung.

Ergänzend werden für Patient*innen mit AD psychoedukative Programme (z. B. Neurodermitisschulungen wie AGNES e. V. Kinder und Jugendliche sowie deren Eltern und ARNE für an AD leidende Erwachsene) empfohlen.

### S2k-Leitlinie „Therapie der Hidradenitis suppurativa/Acne inversa (HS/AI)“ [17]

Die vorbestehende S2k-Leitlinie wurde umfangreich aktualisiert. Aktuelle Daten zur Prävalenz in Deutschland (0,3 %) sowie global (0,40 % , 95 %-KI [Konfidenzintervall]: 0,26–0,63 %) wurden aufgenommen.

Für die Dokumentation der Krankheitsaktivität soll in der täglichen Praxis ein validiertes Klassifikationsinstrument, v. a. das International Hidradenitis Suppurativa Severity Score System (IHS4) verwendet werden. Der Hurley-Score soll für die Dokumentation der Entscheidung einer chirurgischen Therapie eingesetzt werden.

Die Therapieempfehlungen orientieren sich in der neuen Fassung der Leitlinie an einer Einteilung in „entzündliche“ und „vorwiegend nicht entzündliche“ Formen der HS/AI.

Die Kombination einer medikamentösen Therapie zur Reduktion der Entzündung mit einem operativen Verfahren zur Beseitigung des irreversiblen Gewebeschadens wurde als ganzheitliches Therapieverfahren bei HS/AI hervorgehoben. Als besonders wesentliche Veränderung bei aktiver (entzündlicher) HS/AI sind die nun starke (soll) Empfehlung für die systemische Therapie mit Doxycyclin 2‑mal 100 mg/Tag und die nur noch einfache Empfehlung (sollte) für Clindamycin/Rifampicin zu erwähnen. Neu aufgenommen bei aktiver HS/AI mit starker Empfehlung wurde Secukinumab. Zum Zeitpunkt der Veröffentlichung war Bimekizumab noch nicht zugelassen.

Für die nichtentzündliche Form stehen weiterhin verschiedene chirurgische Verfahren zur Verfügung.

### S2k-Leitlinie „Basalzellkarzinom der Haut – Aktualisierung 2023“ [12]

Das Basalzellkarzinom (BZK) ist weiterhin der häufigste bösartige Hauttumor bei hellhäutigen Menschen mit weiterhin steigender Inzidenz. Die aktualisierte S2k-Leitlinie von 2023 betont die Bedeutung einer frühzeitigen Diagnose und einer risikoadaptierten Therapie. Bei Auftreten von multiplen BZK vor dem 20. Lebensjahr soll eine Abklärung zum Ausschluss eines Syndroms (Basalzellkarzinomsyndrom, Bazex-Dupré-Christol-Syndrom, Rombo-Syndrom) erfolgen.

Die chirurgische Exzision bleibt der Goldstandard, insbesondere bei Hochrisiko-BZK (s. Tab. 2 der Leitlinie: Einteilung der Rezidivrisikostufen bei Basalzellkarzinomen). Für lokal fortgeschrittene oder metastasierte BZK bleiben Hedgehog-Inhibitoren wie Vismodegib und Sonidegib die bevorzugte Erstlinientherapie. Neu ist die Zulassung von PD-1-Inhibitoren wie Cemiplimab in der Zweitlinientherapie für Patienten, die auf Hedgehog-Inhibitoren nicht ansprechen oder diese nicht vertragen. Die Leitlinie unterstreicht die Bedeutung einer interdisziplinären Zusammenarbeit und empfiehlt, komplexe Fälle in Tumorkonferenzen zu besprechen. Zudem wird auf die Notwendigkeit regelmäßiger Nachsorgeuntersuchungen hingewiesen, um Rezidive frühzeitig zu erkennen.

### S2k-Leitlinie „Kutane Lyme Borreliose“ [8]

Im Rahmen der Aktualisierung der S2k-Leitlinie zur kutanen Lyme-Borreliose kam es zu keinen gravierenden Änderungen. Bezüglich der immer wieder aufkommenden Diskussion um die prophylaktische Antibiotikaeinnahme nach Zeckenstichen, weist die Leitlinie darauf hin, dass nur bei einem kleinen Teil der Zeckenbisse eine Übertragung stattfindet und auch davon nur ein kleiner Teil der Infizierten erkrankt, sodass von einer prophylaktischen Antibiotikagabe sowohl topisch als auch systemisch weiterhin abgeraten wird. Es wird empfohlen, die Einstichstelle bis zu 6 Wochen zu beobachten, um etwaige Infektionen zu erkennen. Beim klinisch typischen Auftreten eines Erythema migrans soll ohne vorherige Borrelienserologie eine Antibiotikabehandlung, vorzugsweise mit Doxycyclin (bei Kindern ab dem 9. Lebensjahr) oder Amoxicillin, erfolgen. Eine Serologie soll nur bei atypischem klinischem Erscheinungsbild erfolgen.

### S2k-Leitlinie „Diagnostik und Therapie der zirkumskripten Sklerodermie“ [7]

Die aktualisierte Leitlinie zur zirkumskripten Sklerodermie (ZS) enthält bedeutende Neuerungen bezüglich der Klassifikation. Es werden nun 4 Hauptformen unterschieden: limitierte, generalisierte, lineare und gemischte ZS. Die Therapie stützt sich weiterhin auf die topische Therapie (insbesondere mittel- bis hochpotente Glukokortikosteroide), UV-Therapie und systemische Therapien. Neben UVA1- (starke Empfehlung) und PUVA(Psoralen plus UVA)-Therapie (einfache Empfehlung) ist nun auch die Schmalspektrum-UVB-Phototherapie (offene Empfehlung) aufgenommen worden.

Methotrexat bleibt das Mittel der ersten Wahl unter den Systemtherapien. Bei refraktären Verläufen sind Mycophenolat-Mofetil, Mycophenolsäure und Abatacept (alle „off-label“) Optionen der zweiten Wahl.

Neu aufgenommen wurden Empfehlungen zur autologen Fettstammzelltransplantation und Lasertherapie. Zudem wurde der Therapiealgorithmus aktualisiert, um eine moderne und effektive Behandlungsstrategie zu gewährleisten.

### S1-Leitlinie „Analer Pruritus“ [4]

Die aktualisierte S1-Leitlinie zum analen Pruritus berücksichtigt neue wissenschaftliche Erkenntnisse zur Ätiologie und Diagnostik. Sie betont die Bedeutung einer gründlichen Anamnese und körperlichen Untersuchung, um mögliche zugrunde liegende Erkrankungen zu identifizieren (s. Tab. 1 der Leitlinie: Überblick über mögliche Ursachen von analem Pruritus). Die Therapieempfehlungen basieren auf 3 Säulen:

1. Behandlung zugrunde liegender ursächlicher oder aggravierender Erkrankungen,
2. Allgemeinmaßnahmen zur Beseitigung bzw. Minderung irritativer Reize im Analbereich,
3. symptomatische, juckreizlindernde Therapie.

Zudem werden nichtmedikamentöse Maßnahmen wie die Anpassung der Hygiene und Lebensgewohnheiten betont.

### S1-Leitlinie „Diagnostik und Therapie des Analekzems“ [6]

Die aktualisierte S1-Leitlinie zur Diagnostik und Therapie des Analekzems umfasst neue Erkenntnisse zur Ätiologie und zu den Therapiemöglichkeiten. Der Fokus liegt auf einer gründlichen Anamnese und Untersuchung, um mögliche Ursachen wie Allergien oder Infektionen zu identifizieren (s. Tab. 1 der Leitlinie: Übersicht über die wichtigsten Differenzialdiagnosen des Analekzems und wegweisende diagnostische Verfahren). Häufige Allergene beim allergischen Analekzem sind Duftstoffe, Konservierungsmittel, Lokalanästhetika wie Cinchocain und Benzocain, Antibiotika und Antimykotika. Es wird auch darauf hingewiesen, dass auch Nahrungsmittelallergien eine Rolle für die Entstehung und Aufrechterhaltung perianaler Ekzeme bei atopischer Diathese spielen können. Die Therapie basiert auf der Vermeidung irritierender Substanzen, topischen Glukokortikosteroiden und der Auswahl geeigneter Pflegeprodukte (z. B. hydrophile O/W[Öl-in-Wasser]-Zubereitungen). Zusätzlich wird die Rolle von Antihistaminika und Calcineurininhibitoren (ggf. „off-label“ je nach Indikation) bei chronischen Fällen hervorgehoben.

### S1-Leitlinie „Definition und Therapie der primären Hyperhidrose“ [5]

Die aktualisierte S1-Leitlinie zur primären Hyperhidrose definiert die Erkrankung als übermäßiges Schwitzen ohne zugrunde liegende Krankheit.

Therapie der ersten Wahl der axillären Hyperhidrose bleibt weiterhin die topische Anwendung von Antiperspirantien (z. B. Aluminiumchlorid). Als neue Therapieoption wurde hier das topische Anticholinergikum Glycopyrroniumbromid-1 %-Creme ergänzt. Besonders wichtig ist bei dieser neuen Therapie, darauf zu achten, da das Präparat nicht in die Augen kommt, da dort dann eine symptomatische Mydriasis auftreten kann. Daher eignet es sich auch nicht für eine Anwendung bei palmarer Hyperhidrose. Als weitere Optionen für die Behandlung axillärer Hyperhidrose finden eine Behandlung mit Radiofrequenz, Mikrowellen oder Ultraschall, eine systemische Therapie mit Anticholinergika bzw. Neuroleptika oder Psychopharmaka mit anticholinerger Wirkung und die chirurgische Schweißdrüsenentfernung mittels Kürettage, Saugkürettage oder Exzision Erwähnung. Hervorzuheben ist, dass die Iontophorese für die axilläre Anwendung laut Leitlinie nur „eine untergeordnete Rolle spielt“ (aufwendiger, geringere Datenlage zur Wirksamkeit), dafür aber bei palmarer und plantarer Hyperhidrose an zweiter Position erwähnt wird und in dieser Indikation die Wirksamkeit durch klinische Studien gut belegt ist. Systemische Anticholinergika sollten aufgrund möglicher systemischer Nebenwirkungen nur bei Bedarf und zeitlich begrenzt eingesetzt werden.

Chirurgische Verfahren wie die Saugkürettage oder die Sympathektomie sind bei Therapieversagen die letzte Option und sollten wegen ihrer unerwünschten Nebenwirkungen sorgfältig evaluiert werden.

## Ausgewählte deutsche Leitlinien unter gemeinsamer Federführung mit der Deutschen Dermatologischen Gesellschaft (DDG) bzw. Federführung durch andere Fachgesellschaften

### S3-Leitlinie „Aktinische Keratose und Plattenepithelkarzinom (PEC) der Haut“ [3]

Die Therapie der aktinischen Keratose wurde aktualisiert und um neu zugelassene Präparate (Kaliumhydroxid 5 %-Lösung, 5‑Fluorouracil 4 %-Creme, photodynamische Therapie mit Aminolävulinsäure (ALA-cPDT: ALA-Nanoemulsion), Tirbanibulin) ergänzt. Neu formuliert wurden Empfehlungen zur Cheilitis actinica sowie zum Morbus Bowen.

Für die chirurgische Therapie des kutanen PEC wurden die Empfehlungen zur Methodenauswahl aktualisiert. Je nach Vorliegen klinischer Risikofaktoren – wie etwa ungünstige Lokalisation (z. B. Ohr, Lippe, Schläfe), Lokalrezidiv, Durchmesser > 2 cm, Immunsuppression oder fehlende Verschieblichkeit vom Untergrund – sowie histologischer Risikofaktoren (Eindringtiefe > 6 mm, Desmoplasie, perineurale Invasion, Überschreiten der Subkutis oder schlechte Differenzierung [G3]) wird nun zwischen 2 Vorgehensweisen unterschieden:

- *Exzision mit histologischer Kontrolle* bei Tumoren ohne oder mit wenigen Risikofaktoren. Ziel ist die vollständige Entfernung (R0) mittels konventioneller Exzision und nachfolgender serieller Schnittrandkontrolle.
- *Mikrographisch kontrollierte Chirurgie (MKC)* wird empfohlen bei Vorliegen eines oder mehrerer klinischer oder histologischer Risikofaktoren. Diese Methode erlaubt eine knappe Exzision mit lückenloser Kontrolle aller Resektionsränder, um R0 sicherzustellen.

In der deutschen S3-Leitlinie wird aufgrund fehlender Evidenzbasis kein verbindlicher Wert für die Größe des Sicherheitsabstands definiert; stattdessen erfolgt die Empfehlung anhand der Risikofaktoren und des gewählten Vorgehens.

Des Weiteren wurden die Empfehlungen zur Systemtherapie aktualisiert (Änderungen der Zulassung von Cemiplimab vom 08/2019), und auf Alternativen nach Progress und bei Kontraindikationen wurde eingegangen. Ein Abschnitt zur Palliativmedizin wurde ergänzt, und die Empfehlungen zur Nachsorge wurden angepasst.

### Deutsch-Österreichische S2k-Leitlinie „Anale Dysplasien und Analkarzinom-Screening bei Menschen mit humanem Immundefizienz-Virus (HIV)“ [2]

Menschen mit HIV haben ein bis zu 100-fach erhöhtes Risiko für Analkarzinome. Daher wird in der aktualisierten Leitlinie (Vorversion von 2015) ein Screening für Vorläufer des Analkarzinoms für Menschen mit HIV ab dem 45. Lebensjahr empfohlen, für Männer, die Sex mit Männern haben und mit HIV leben, sogar bereits ab dem 35. Lebensjahr. Für weitere Risikogruppen (beispielsweise Frauen, ungeachtet des HIV-Status, mit hochgradigen vulvären Dysplasien) wurde ebenfalls eine Screeningempfehlung formuliert. Werden Vorläufer des Analkarzinoms gefunden, kann eine Therapie (vorzugsweise mit Elektrokaustik/Argonplasmakoagulation, Trichloressigsäure oder Exzision/operative Abtragung) durchgeführt werden, und das Risiko für Analkarzinome wird signifikant reduziert. Das Screening startet mit einer klinischen Untersuchung plus analer Zytologie via Abstrich. Gegebenenfalls folgt eine Zuweisung zur hochauflösenden Anoskopie, mit der Dysplasien diagnostiziert und im Anschluss therapiert werden können.

### S2k-Leitlinie „Diagnostik und Therapieoptionen von Aphthen und aphthoiden Läsionen der Mund- und Rachenschleimhaut“ [1]

Bei den rezidivierenden Aphthen liegt ein multifaktorieller ätiologischer Hintergrund vor. Aphthen und aphthoide Läsionen der Mund- und Rachenschleimhaut sind in jedem Fall von oralen potenziell malignen Erkrankungen (OPMD) abzugrenzen. Differenzialdiagnosen sind Malignome und deren Vorstufen, andere Stomatopathien, reaktive Veränderungen der Mund- und Rachenschleimhaut, gastrointestinale Syndrome, mukokutane Erkrankungen des rheumatischen Formenkreises, bullöse und lichenoide Dermatosen und Infektionskrankheiten. Die Therapie von chronisch rezidivierenden oropharyngealen Aphthen zielt auf die Schmerzlinderung, ein Nachlassen der aphthös bedingten funktionellen Einschränkungen, eine Reduzierung der Häufigkeit der Aphthenschübe und eine Abnahme des Schweregrades der Rezidive ab. Bei habituellen Aphthen stellen topisch anzuwendende Präparate die Therapie der Wahl dar. Dabei kommen Präparate zum Einsatz, die adstringierend, antiseptisch, antiinflammatorisch, lokalanästhetisch oder antibiotisch wirken:

- *Stufe 1*: lokale filmbildende Präparate oder Sucralfat („off-label“), bei Major-Aphthen zusätzlich wiederholte tägliche Mundspülungen mit 5 % Tetracyclin (ab dem 8. Lebensjahr) wegen ihrer antibakteriellen Eigenschaften und der Inhibition der zum Entzündungsprozess beitragenden Matrixmetalloproteinasen.
- *Stufe 2*: Triamcinolonacetonid (0,1 % Haftsalbe).
- *Stufe 3*: Bei therapieresistenten oder schweren Formen wird eine Überweisung an ein Fachzentrum empfohlen. Zudem kann die Einleitung einer Systemtherapie mit Colchicin („off-label“) versucht werden.
- *Stufe 4:* Zur Akutintervention (zeitlich begrenzt) kann Prednisolon eingesetzt werden, bei Nichtansprechen von therapieresistenten, schweren Aphthosen auf Colchicin („off-label“) kann Dapson oder Apremilast bei Behçet-Syndrom eingeleitet werden. Bei Ineffektivität oder unerwünschten Arzneiwirkungen können im Einzelfall auch Azathioprin, Ciclosporin A, Dapson, ein TNF(Tumor-Nekrose-Faktor)-α-Inhibitor oder Interferon α erwogen werden („off-label“ je nach Indikation).

Alternativ zur topischen oder systemischen Anwendung von Medikamenten werden auch beim Einsatz eines Kohlenstoffdioxid(CO_2_)-Lasers, Dioden- oder Neodym-dotierten Yttrium-Aluminium-Granat-Lasers (Nd:YAG-Laser) eine rasche bzw. nach wenigen Tagen merkliche Schmerzreduktion sowie ein schnellerer Heilungsverlauf beobachtet.

### S2k-Leitlinie „Allergologische Diagnostik von Überempfindlichkeitsreaktionen auf Arzneimittel“ [10]

Arzneimittelüberempfindlichkeitsreaktionen unter dem Bild einer Urtikaria/einer Anaphylaxie, die innerhalb von 1(–6) h nach Arzneimittelzufuhr („Sofortreaktionen“) oder als Exanthem mehrere Stunden bis Tage später („Spätreaktionen“) auftreten, sollen allergologisch abgeklärt werden. Durch geeignete allergologische Diagnostik soll versucht werden, eine Beteiligung des Immunsystems (Allergie) nachzuweisen.

Es wird empfohlen die allergologische Diagnostik durchzuführen – einerseits, um gefährdete Patient*innen rechtzeitig und eindeutig zu erkennen, andererseits, um ungerechtfertigte Einschränkungen in der Arzneimitteltherapie zu verhindern. Die allergologische Diagnostik sollte innerhalb von 4 Wochen bis 6 Monaten nach der Reaktion angestrebt werden. Die Einordnung einer Arzneimittelreaktion und damit die Planung der Diagnostik sollen sich nach dem klinischen Bild, dem zeitlichen Ablauf und dem vermuteten ursächlichen Arzneimittel richten. Bei eindeutig positivem Befund eines validierten Haut- und/oder Labortests in Verbindung mit einer dazu passenden Anamnese kann/können der/die Auslöser als ausreichend gesichert angesehen werden. Ist nach der Haut- und Labordiagnostik keine Diagnose möglich, sollte nach Nutzen-Risiko-Abwägung eine kontrollierte Provokationstestung angestrebt werden. Das Ergebnis der allergologischen Diagnostik sollte dem/der Patient*in ausführlich erklärt werden. Eine diagnostizierte allergische oder nichtallergische Überempfindlichkeit auf ein oder mehrere Arzneimittel soll in einem Allergiepass dokumentiert werden, um sicherzustellen, dass auf diese(s) Arzneimittel zukünftig verzichtet wird – im Einzelfall können, soweit bekannt und als sinnvoll erachtet, ergänzend auch vertragene alternative Arzneimittel im Allergiepass genannt werden.

### S2k-Leitlinie „Diagnose und Therapie der Bienen- und Wespengiftallergie“ [14]

Bei einem Hymenopterenstich wird Gift in die Haut injiziert, das bei sensibilisierten Individuen allergische Reaktionen hervorrufen kann. Etwa 3 % der Bevölkerung entwickeln eine systemische allergische Reaktion (SAR) nach einem Stich.

Diese Leitlinie beschreibt das diagnostische und therapeutische Vorgehen bei SAR. Nach einer verstärkten lokalen Reaktion ist meist nur eine symptomatische Behandlung nötig, keine spezifische Diagnostik oder Immuntherapie.

Bei SAR sollen Risikofaktoren erfasst werden, etwa durch Beruf oder Hobby, Mastzellerkrankungen und unkontrolliertes Asthma v. a. bei Kindern.

Daher sollen ab einer über die Haut hinausgehenden SAR (gemäß Klassifikation nach Ring und Messmer > Schweregrad I) zur Erfassung einer möglichen Mastozytose eine Bestimmung der basalen Serumtryptase und eine Hautinspektion erfolgen.

Der Nachweis einer Hymenopterengift(HG)-Sensibilisierung erfolgt mit der Bestimmung von spezifischen Ig(Immunglobulin)E-Antikörpern (sIgE) gegen Bienen- und/oder Wespengift. Hauttests können unterbleiben, wenn mit In-vitro-Verfahren bereits eine eindeutige Diagnose gestellt werden kann. Falls weder Labortests noch Hauttests zu einer Therapieentscheidung führen, können zelluläre Tests hilfreich sein.

Die Behandlung umfasst Expositionsprophylaxe, Selbsthilfemaßnahmen und eine HG-Allergen-Immuntherapie (HG-AIT). Bei einer SAR Grad I und ohne Risikofaktoren für schwerere Reaktionen ist weder ein Adrenalin-Autoinjektor noch eine HG-AIT erforderlich. In bestimmten Fällen, etwa bei zusätzlichem Risikofaktor oder Lebensqualitätseinschränkung, kann eine HG-AIT sinnvoll sein. Betablocker und Angiotensin-Converting-Enzyme-Hemmer stellen keine Kontraindikation dar, sollten aber mit den Patienten besprochen werden.

Für die HG-AIT soll der Giftextrakt verwendet werden, der gemäß Anamnese und Diagnostik als ursächlich ermittelt wurde. Eine HG-AIT kann bei Patienten ohne besondere Risikofaktoren nach 3 bis 5 Jahren beendet werden, wenn die Erhaltungstherapie vertragen wurde. In Fällen mit hohem Risiko, etwa bei Mastozytose oder schweren Reaktionen, kann eine längere Therapie erwogen werden. Bei Patienten mit SAR Grad III oder höher oder weiteren Risikofaktoren wird empfohlen, ein Notfallset mit Adrenalin-Autoinjektor (AAI) mitzuführen, auch nach Ende der HG-AIT.

Im Jahr 2023 wurden von der DDG bzw. mit Beteiligung der DDG weitere Leitlinien veröffentlicht, darunter:

- S3-Leitlinie: Lokaltherapie schwerheilender und/oder chronischer Wunden,
- S2k-Leitlinie: Diagnostik, Prävention und Therapie des Handekzems,
- S1-Leitlinie: Diagnostik und Therapie der Necrobiosis lipoidica,
- S2k-Leitlinie: Lipödem,
- S2k-Leitlinie: Diagnostik und Therapie des Ulcus cruris venosum.

## Fazit für die Praxis

- Leitlinien werden kontinuierlich aktualisiert und an das medizinische Wissen angepasst.
- Aufzufinden sind die deutschen Leitlinien unter www.awmf.org.
- Jede Leitlinienempfehlung sollte unter Berücksichtigung der individuellen Situation des Patienten sorgfältig geprüft, angepasst und bei Bedarf modifiziert werden.
